# Management and Repair of a Large Basal Cell Carcinoma With Associated Varicosities Draining Into the Right Internal Jugular Vein

**DOI:** 10.7759/cureus.88999

**Published:** 2025-07-29

**Authors:** Alexander M Hammond, Neelesh P Jain, Sailesh Konda

**Affiliations:** 1 Department of Dermatology, University of Florida College of Medicine, Gainesville, USA

**Keywords:** basal cell carcinoma (bcc), dermatology and dermatologic surgery, mohs micrographic surgery (mms), vegf angiogenesis, xenograft

## Abstract

Basal cell carcinoma (BCC) is the most prevalent skin cancer and carries a favorable prognosis when treated early. Mohs micrographic surgery (MMS) is the standard of care for large BCCs greater than 2 cm on the trunk or extremities, given its ability to achieve a high cure rate while maximizing preservation of healthy tissue. While arborizing telangiectasias are a hallmark feature, locally advanced tumors may demonstrate more obvious varicosities that warrant more rigorous preoperative planning. Herein, we present a case of a large BCC on the upper back with pronounced varicosities draining into the right internal jugular vein treated with MMS. Preoperative imaging and clinical evaluation were important for ensuring treatment success in this locally advanced BCC. The tumor cleared in one stage, and a 14-month follow-up revealed no tumor recurrence and regression of the aberrant vascularization.

## Introduction

Basal cell carcinoma (BCC) is the most prevalent form of skin cancer, typically characterized by its slow growth rate and low metastatic potential [1]. BCCs generally have a favorable overall prognosis when treated early. Large or locally advanced BCCs, estimated to be 0.8% of all cases, can present unique challenges, particularly when they exhibit unusual patterns of vascularization beyond the classic arborizing telangiectasias routinely observed on dermoscopy [2]. Mohs micrographic surgery (MMS) is the treatment of choice for large BCCs (>2 cm) or BCCs in cosmetically or functionally sensitive areas. MMS offers the benefit of confirming histologic tumor clearance on the same day as surgery while maximizing preservation of healthy tissue. Herein, we present a case of a large BCC on the upper back with superficial angiogenesis of local veins presenting as varicosities draining into the right internal jugular vein treated with MMS.

## Case presentation

**Figure 1:**
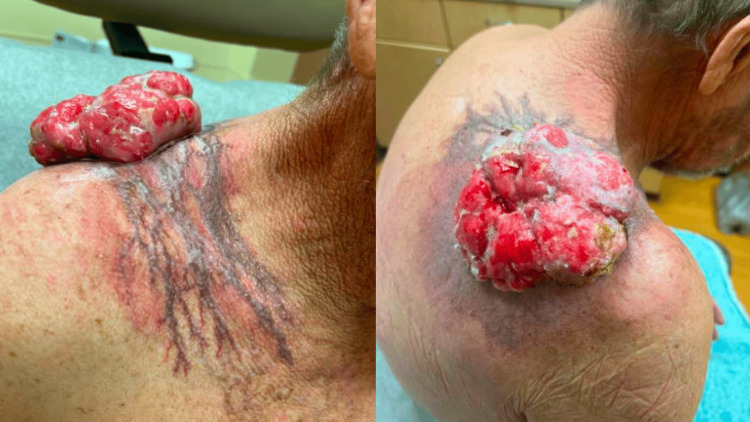
A 9.0 x 9.0 cm, pink, exophytic, ulcerated tumor on the right upper back surrounded by superficial, blue, reticular varicosities extending from the right upper back to the right upper chest.

A 65-year-old man presented with a 9.0 x 9.0 cm, pink, exophytic, ulcerated tumor on the right upper back surrounded by superficial, blue, reticular varicosities extending from the right upper back to the right upper chest (Figure 1).

**Figure 2:**
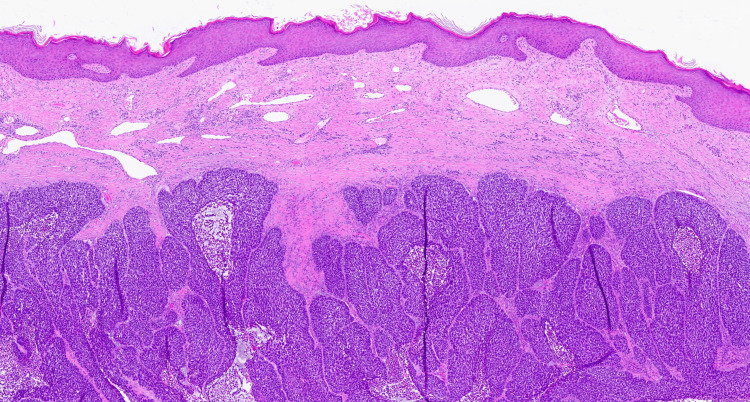
Extending from the undersurface of the epidermis are atypical basaloid nests of cells. At the periphery of the nests, there is a palisaded arrangement with mitotic figures and occasional necrotic cells present.

**Figure 3:**
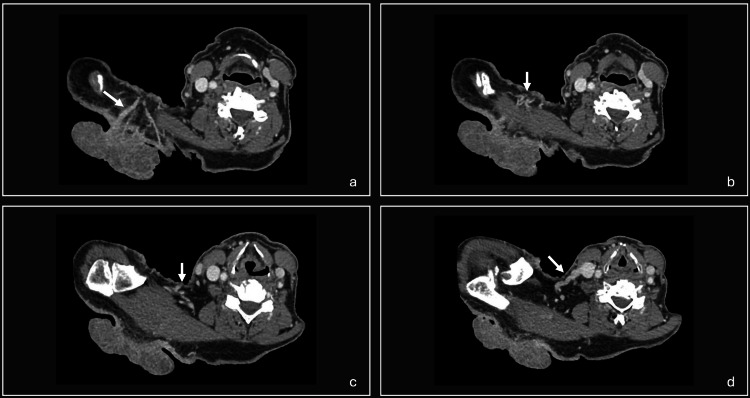
CT of the neck with IV contrast. (a) A robust vascularization with large draining veins extending from the lesion. (b and c) Draining veins from the lesion pooling in a central venous confluence. (d) Draining veins from the lesion extending to the right internal jugular vein.

Over the past 10 years, the tumor gradually enlarged, and the patient began experiencing bleeding from the lesion as well as increasing tenderness. Shave biopsy revealed a nodular basal cell carcinoma (Figure 2). A CT scan with IV contrast of the neck and chest revealed robust vascularization with large draining veins extending from the lesion to the right internal jugular vein and a notable absence of distant metastases or invasion into the muscle or bone (Figure 3).

**Figure 4:**
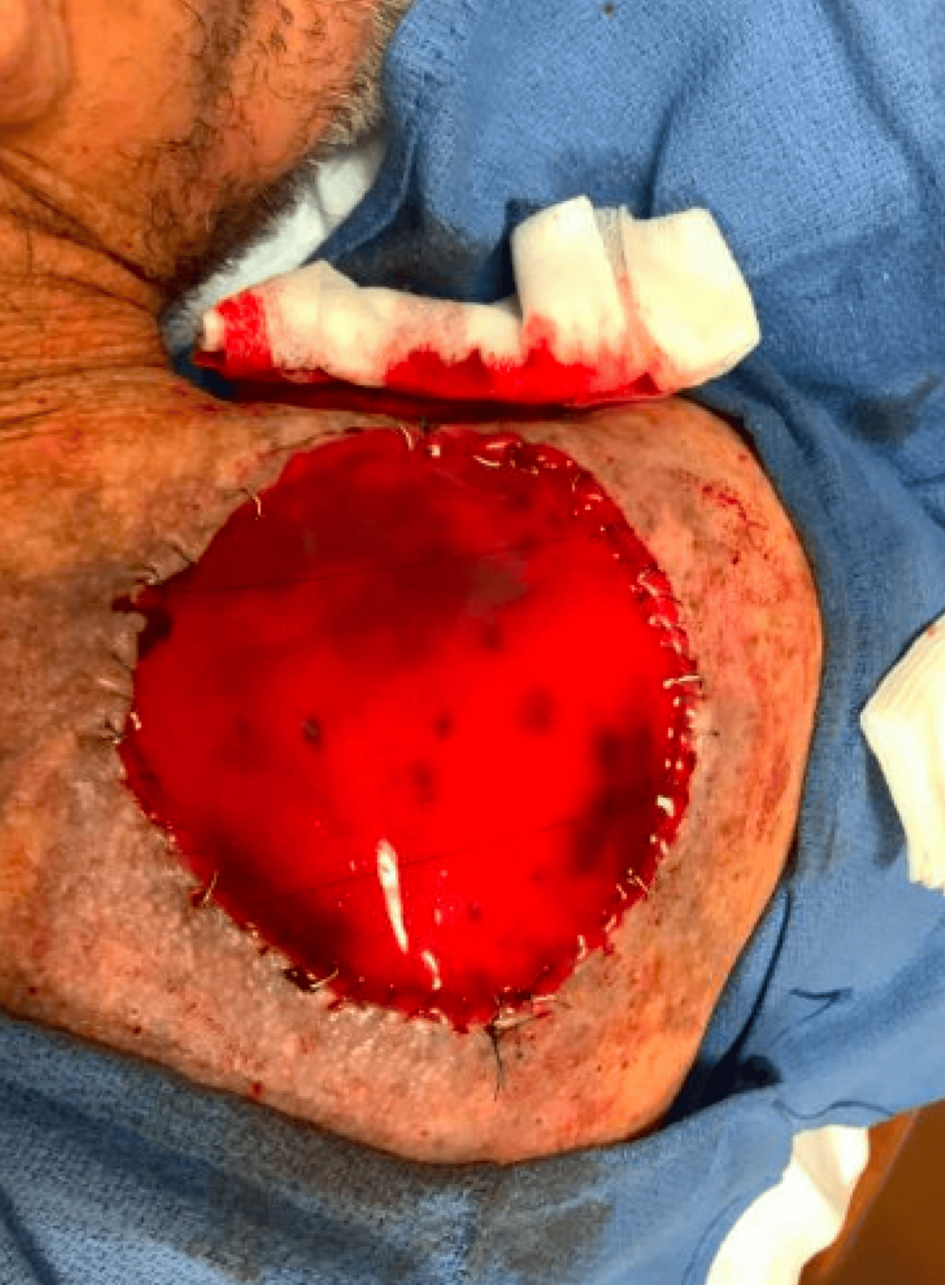
Primary postoperative defect with placement of a dermal repair scaffold xenograft stapled into place.

Our patient was offered treatment with MMS with or without adjuvant immunotherapy versus immunotherapy alone. The patient opted for treatment with MMS alone, and the tumor cleared in one stage. There was no evidence of tumor extension beyond the subcutaneous fat. Given the size of the tumor and the proximity of large outflow vasculature, 180 mL of 0.1% tumescent local anesthesia (TLA) was utilized. The postoperative defect measured 10.2 x 10.0 cm and was allowed to heal via second intention with placement of a dermal repair scaffold xenograft (Integra®, Integra LifeSciences, Princeton, NJ) (Figure 4).

**Figure 5:**
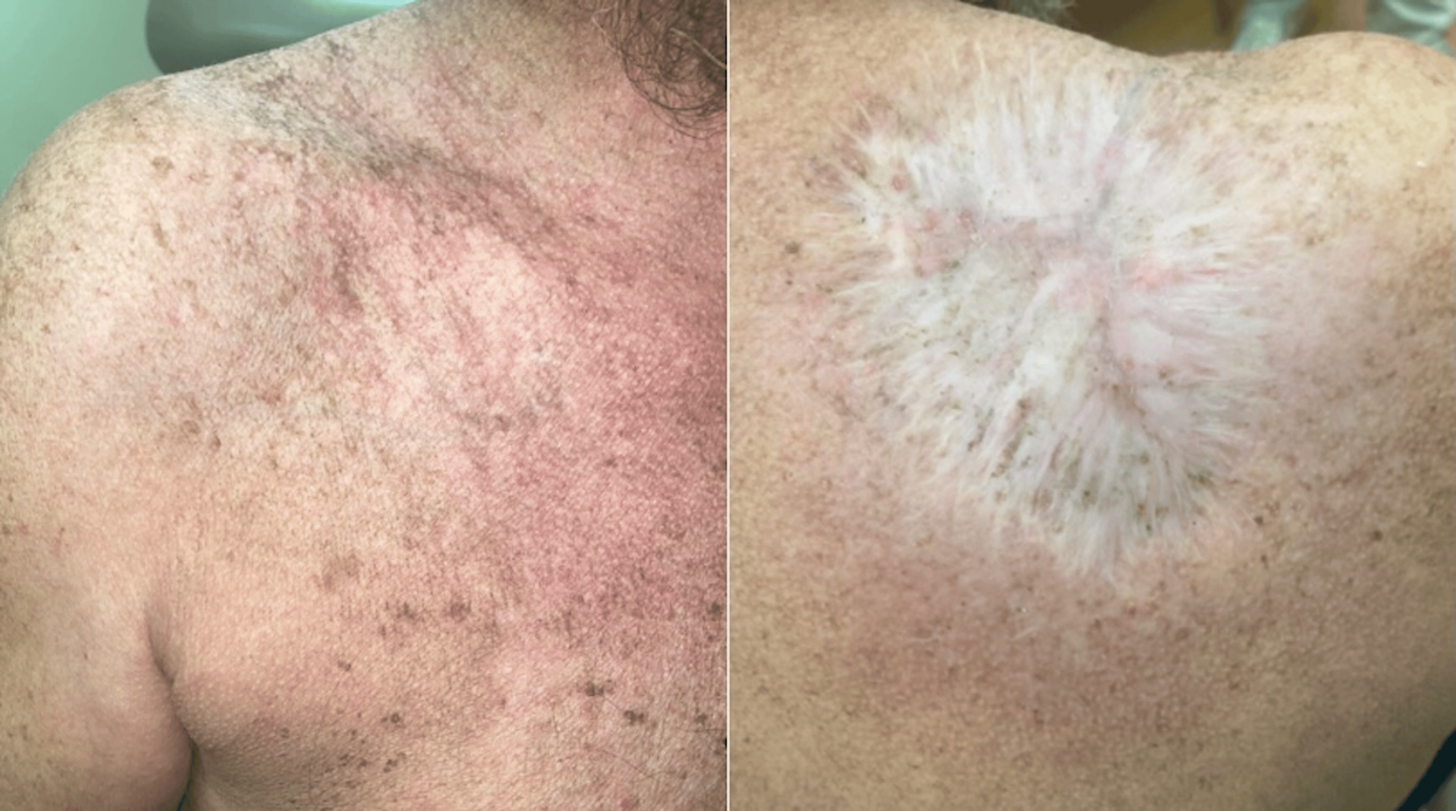
Fourteen months postoperative images showing a pink, atrophic scar with significant fading of the surrounding superficial varicose veins.

Over the course of 14 months, the postoperative defect healed with a pink, atrophic scar along with significant fading of the surrounding superficial varicose veins (Figure 5).

## Discussion

This large BCC exhibited pronounced tumor-driven angiogenesis, resulting in dilated veins draining directly into the internal jugular vein, highlighting a unique vascular pattern not previously reported. Angiogenesis is crucial for the proliferation of tumors, as evidenced by the increased expression of vascular endothelial growth factor (VEGF) in more than half of BCCs and in adjacent non-tumoral cells [3]. VEGF acts on endothelial cells to promote blood vessel growth to provide nutrients and oxygen for the tumor. In addition to its angiogenic effects, VEGF also induces skin carcinogenesis by acting on keratinocytes directly; studies have shown that the loss of VEGFR-1 on keratinocytes prohibits tumor proliferation [4].

While arborizing vessels and short, fine telangiectasias are dermoscopic hallmark features of BCC [5], clinically apparent varicosities arising from a BCC are a rare entity. There is no reported evidence that the degree of aberrant vasculature in a BCC affects prognosis. Around 1% of BCCs are considered locally advanced BCC (LABCC), where a multidisciplinary approach to treatment should be considered [2]. After an extensive discussion on the risks, benefits, and alternatives to treatment with MMS with or without adjuvant immunotherapy, our patient elected to treat solely with MMS, which achieved tumor clearance in one stage. Given the large tumor size, continued surveillance was recommended, even though tumor staging was Brigham and Women’s Hospital T1 with a low risk of metastasis and death [6].

The tumor-derived angiogenesis seen in this case was considered during preoperative planning with efforts to minimize bleeding and limit systemic absorption of local anesthesia. TLA offers several benefits, including prolonged anesthesia, reduced bleeding, and reduced risk of lidocaine toxicity [7]. Repair of the resulting large surgical defect also required maintenance of the function of the patient’s underlying musculature and nearby acromioclavicular joint. The result achieved via second intention healing with placement of a skin substitute was satisfactory (Figure 5). The VEGF-induced superficial vasculature regressed after surgery, and the hemosiderin deposition in the skin eventually faded.

## Conclusions

After 14 months of follow-up, our patient is pleased with the aesthetic and functional result of the xenograft and has no signs of tumor recurrence. This case underscores the importance of recognizing aberrant angiogenesis in BCCs and the need for meticulous pre-surgical planning to achieve tumor clearance with MMS such that no complications related to critical local vasculature arise. While BCCs have a favorable prognosis, the presence of atypical vascularization necessitates a nuanced approach to management and surveillance. Pre-surgical imaging and the use of TLA helped to reduce the risk of complications. Continued research into the biological behavior of these tumors will enhance our understanding and inform future therapeutic strategies.
